# Vector and position coding in goal-directed movements

**DOI:** 10.1007/s00221-016-4828-9

**Published:** 2016-11-17

**Authors:** Marieke C. W. van der Graaff, Eli Brenner, Jeroen B. J. Smeets

**Affiliations:** Department of Human Movement Sciences, Vrije Universiteit Amsterdam, Amsterdam, The Netherlands

**Keywords:** Human, Arm movement, Precision, Variability, Reference frame

## Abstract

Two different ways to code a goal-directed movement have been proposed in the literature: vector coding and position coding. Assuming that the code is fine-tuned if a movement is immediately repeated, one can predict that repeating movements to the same endpoint will increase precision if movements are coded in terms of the position of the endpoint. Repeating the same movement vector at slightly different positions will increase precision if movements are coded in terms of vectors. Following this reasoning, Hudson and Landy (J Neurophys 108(10):2708–2716, 2012) found evidence for both types of coding when participants moved their hand over a table while the target and feedback were provided on a vertical screen. Do we also see evidence for both types of coding if participants repeat movements within a more natural visuo-motor mapping? To find out, we repeated the study of Hudson and Landy (J Neurophys 108(10):2708–2716, 2012), but our participants made movements directly to the targets. We compared the same movements embedded in blocks of repetitions of endpoints and blocks of repetitions of movement vectors. Within blocks, the movements were presented in a random order. We found no benefit of repeating either a position or a vector. We subsequently repeated the experiment with a similar mapping between movements and images to those used by Hudson and Landy and found that participants only clearly benefit from repeating a position. We conclude that repeating a position is particularly useful when dealing with unusual visuo-motor mappings.

## Introduction

There are many possible ways to move ones hand towards an object, but humans tend to move in a specific way (Morasso 1983). The biomechanics of the arm are very complicated, with many muscles contributing to any goal-directed arm movement. Many theories of motor control therefore assume that goal-directed movements are initially planned in some relatively simple higher-level manner and that this plan is then converted into precise motor commands. Two hypotheses about how these movements are planned have received much attention: that they are planned in terms of the direction and distance towards the target (vector coding), and that they are planned in terms of the desired position of the hand or configuration of the arm (position coding).

There is ample evidence in the literature for both vector coding (Bock and Eckmiller 1986; Desmurget et al. 1998; Flanders et al. 1992; Georgopoulos et al. 1981; Gordon et al. 1994; Messier and Kalaska 1997; Rossetti et al. 1995; Vindras et al. 1998) and position coding (Berkinblit et al. 1995; Graziano et al. 2002; McIntyre et al. 1997, 1998; Soechting et al. 1990; van den Dobbelsteen et al. 2001; Thaler and Todd 2009). Thus, some movements may rely on vector coding and others on position coding, or all movements could rely on a combination of the two types of coding (de Grave et al. 2004; Ghez et al. 2007; Scheidt and Ghez 2007; Schenk 2006; van der Graaff et al. 2014). What determines how movements are coded?

One could assume that the combination of codes that gives the most precise movement will be used. If so, and if a code becomes more precise when it is repeated over trials, a code that is repeated will contribute more to the movement. Hudson and Landy (2012) tested this hypothesis by asking participants to make hand movements towards targets on a table while they saw the targets and the feedback on a computer screen in front of them. The movements were presented in different blocks, either consisting of movements towards a certain target position from different directions (to reveal benefits incurred by improving the coding of the final position) or by repeatedly moving the same distance in the same direction between different positions (to reveal benefits incurred by improving the coding of the movement vector). They found that the shapes of the endpoint distributions for identical starting positions and targets were different for the two blocks and concluded that movements were coded both in terms of positions and in terms of vectors. As the visual information was provided on a vertical screen while the movements were made on a table, participants had to learn a new visuo-motor mapping. The finding that movements were coded both in terms of positions and in terms of vectors could therefore have been influenced by having to learn this new mapping.

Do we also see evidence for both of these types of coding if participants do not have to learn a new mapping? To find out, we repeated the study of Hudson and Landy (2012), but our participants saw the targets and feedback on the table on which they made the movements.

## Methods

### Participants and experimental setup

**Fig. 1:**
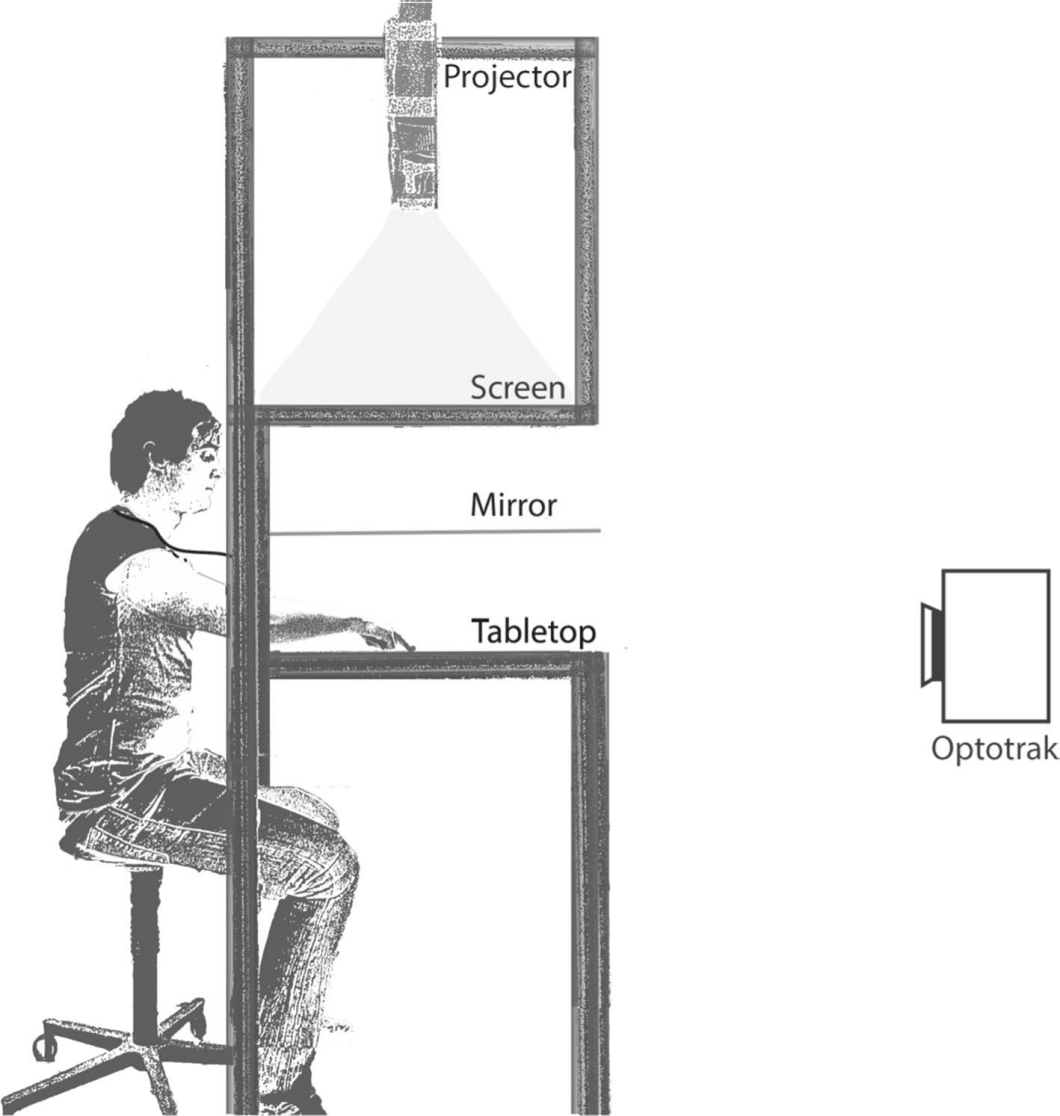
Experimental setup. Participants made movements on the tabletop to targets that they saw reflected in the mirror

Twelve right-handed participants took part in the experiment, which is part of a programme that has been approved by the ethics committee of the faculty of Human Movement Sciences. All participants signed an informed consent form before participating in the study. They were not informed about the purpose of the study. The participants were seated in front of a mirror setup (Fig. 1). In this setup, targets were projected on a horizontal screen above a mirror. The participants could see the targets reflected by the mirror, as if they were on the tabletop below the mirror. The participants moved their invisible hand across this tabletop. Data were recorded with an Optotrak 3020 system at a sampling rate of 200 Hz.

### Procedure

At the start of the experiment, the participants moved with their right index finger to a blue dot (1 cm diameter) at the starting position. They could not see their finger, but received veridical feedback about its position whenever it was less than 5 cm from the starting position. The feedback was provided in the form of a white dot (5 mm diameter) that exactly followed the position of the participant’s finger. When the participants had accurately placed the finger at the starting position (within 2 mm of it for more than 100 ms), the feedback of the finger and the starting position disappeared, and 100 ms later, the dot reappeared at the target position and turned green. The participants were instructed to lift their finger and move as accurately as possible to the target position, with a movement that was not to take more than 300 ms. They could start moving any time after a beep that was played 50 ms after the target appeared. The endpoint of the movement was defined as the position at the moment that the speed was below 7.5 cm/s (movement onset was defined by the same velocity threshold). Once participants finished their movement, a static red dot appeared, providing feedback about the endpoint of the movement. Using the above-mentioned velocity threshold to determine the end of the movement meant that the hand moved another millimetre after the endpoint (and thus feedback) was determined. This static red dot remained visible until the participant’s finger was at the next start position. An additional white dot appeared when participants’ fingers were within 5 cm of the next start position. If the movement took longer than 300 ms, the notification “too slow” was displayed (and a sound was played) to encourage the participants to make faster movements.

**Fig. 2:**
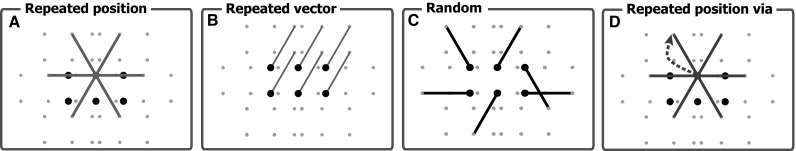
Experimental blocks in which the combinations of start and target positions are presented. **a** Repeated position block, showing all 6 start–target combinations for one of the 6 target positions. **b** Repeated vector block, showing all 6 start–target combinations for one of the six vectors. **c** Random block, showing a possible sequence of six of the 36 start–target combinations. **d** Repeated position via block, showing one path to a start position (*dashed curve*)

We used 6 different target positions (row spacing 6.4 cm, column spacing: 6.8 cm), each combined with 6 different starting positions that were distributed uniformly in directions separated by 60° intervals and were always 11.8 cm from the target. This resulted in 36 start–target combinations (Fig. 2). Each start–target combination was repeated 12 times. We ordered the 12 repetitions of the 36 combinations of start and target positions in three ways within four blocks of trials: a repeated position block, a repeated vector block, a random block and a repeated position with curved trajectory block (repeated position via block). In the repeated position block, participants made sets of 72 movements towards one target, while the starting position (and thus the direction of the movement) varied across trials. The starting position was chosen semi-randomly: the six possible starting positions were each chosen once in random order, after which the six starting positions were each presented again, and so on, with the additional provision that the starting position was never the same on consecutive trials (when switching to a new group of six starting positions). After a set of 72 trials for one target, the experiment continued with a set of 72 trials to the next target, without any notice. This was repeated until all six targets had been tested (432 movements). The repeated vector block was designed in a similar way, but in this block participants repeated the same movement vector towards different targets 72 times before switching to another vector. The target was chosen at random from the six possible values, with the provision that it was never the same on two consecutive trials.

The two blocks described in the previous paragraph correspond to the two conditions of Hudson and Landy (2012). We added two more blocks. In the random block, we never repeated either the position or the vector in two subsequent trials. Thus, after every movement, a subsequent movement was chosen that neither involved the same vector nor moving to the same endpoint. This block provides a baseline for performance without successive repetitions. The fourth block was introduced because we realized that in the repeated position block, the movement from a start position to the target is the opposite of the preceding movement to that start position. Participants might use this to learn the movement vector. To check whether this has an effect, we included the repeated position via block, in which the participants were guided towards the start position along a curved trajectory. Instead of dots indicating the start position, participants saw an array of 12 identical vectors at the top of the screen. These vectors indicated the distance and direction (i.e. vector) between their right finger and the starting position. However, the 12 vectors were initially misdirected by 90°, randomly in a clockwise or counter-clockwise direction in different trials. The magnitude of the misdirection decreased in proportion to the distance moved, guiding the finger to the target along a curved trajectory. When the finger was within 5 cm of the start position, the vectors disappeared and a representation of the participant’s finger position appeared so that the participant could finish the movement towards the starting position under visual guidance.

At the start of the experiment, participants did a practice session of 25 movements (arranged as in the random block), after which they did two blocks of 432 movements. On a different day, they did another practice session of 25 movements, and then the two remaining blocks of 432 movements. The order of the blocks was counterbalanced across participants. Each block took about 30 min. At the start of every block, the coordinates of the Optotrak were aligned to the coordinates of the projector.

### Data analysis

Movement endpoints were defined as the moment the velocity was below 7.5 cm/s. Trials that ended further than 4 times the standard deviation from the mean of the trials for the same start–target combination were excluded from further analysis. This was the case in 1% of the trials. As we were only interested in the variability, we removed any systematic errors by subtracting the mean error for each combination of starting position and target position from each of the 12 trials with that combination of positions. This allowed us to determine measures of variability across different combinations of positions without having to consider possible systematic differences between the endpoints for the different combinations. Irrespective of the way the trials were presented in the different blocks, we grouped movements for the analysis in two different ways: either as 6 groups of 72 movements with the same endpoints (position grouping), or as 6 groups of 72 movements with the same direction from start to target (vector grouping). As we were interested in how having previously made a similar movement (in terms of the two kinds of coding) influences the subsequent precision, the first 24 trials of each sequence of 72 trials were not considered when determining the variability.

We have two predictions that we will test. The first is that if participants benefit from repeating the same endpoint, the variability in endpoints will be lower in the repeated position block than in the random block. We therefore calculated the area within the 95% confidence ellipse for each endpoint (position-grouped) as a measure of precision (for each participant). We compared the average surface areas of such ellipses in the repeated position block with those in the random block with a paired *t* test. The second prediction is that if participants benefit from repeating the same vector, we expect them to be less variable in the direction of their movement (we do not expect a reduced variability in the movement distance, as this was the same in all blocks). We therefore calculated the standard deviation in the endpoints in the direction orthogonal to the movement direction for the vector-grouped block and for the random block. We compared the averages of participant’s standard deviations for the repeated vector block with their averages in the random block with a paired *t* test.

## Results

**Fig. 3:**
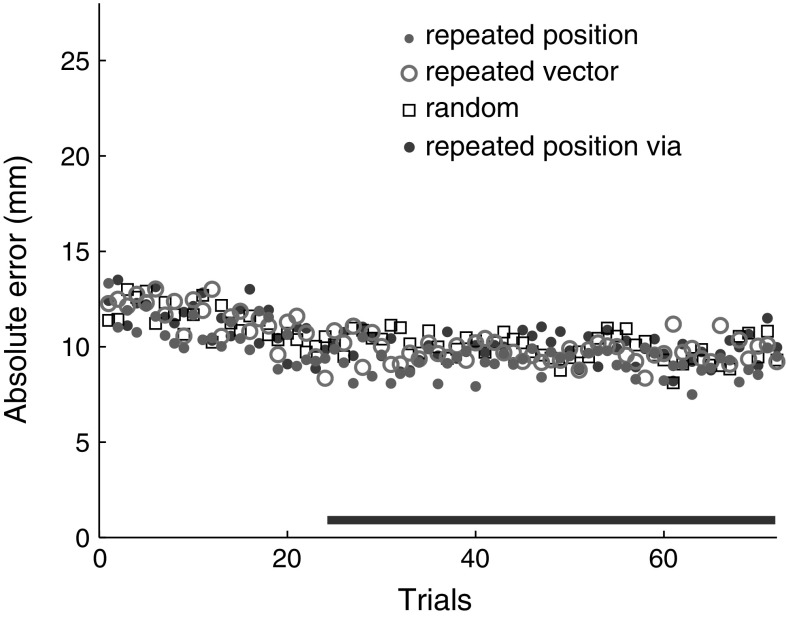
Development of absolute errors during sets of 72 reaches, averaged across sets and participants. The *horizontal bar* indicates the set of trials that was used to determine the variability

On average, the movement times were 268 ms, but they depended on the block [*F*(3,33) = 4.42, *p* < 0.05]. Post hoc comparisons revealed that movement times in the repeated position via block were shorter than in the repeated position and the repeated vector block: 246 ms versus 269 and 286 ms (*p* < 0.05 and *p* < 0.01, respectively). Figure 3 shows the absolute errors on successive trials within each set of 72 trials, averaged across sets and participants. This figure suggests that any refinements to the movements occurred within the first 24 trials.

**Fig. 4:**
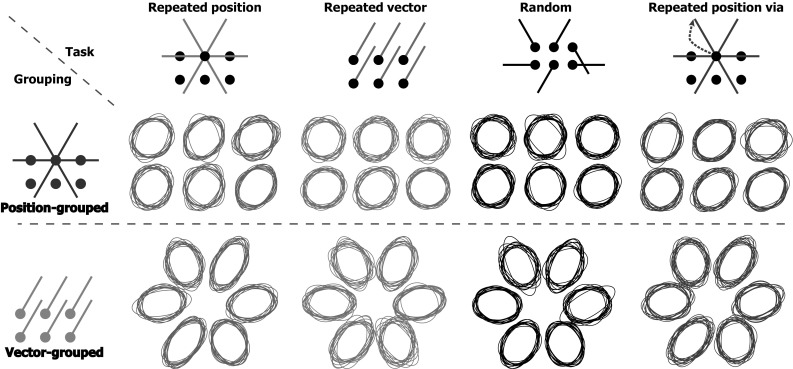
Normalized 95% confidence ellipses for movement endpoints in the different blocks (*columns*) and for the different types of grouping for the analysis (*rows*). *Each ellipse* represents one participant. For the position-grouped endpoints, one ellipse was calculated for every target. For the vector-grouped endpoints, one ellipse was calculated for every movement direction. The surface areas of the ellipses were normalized to illustrate the similarity in shape across participants

Figure 4 shows the shapes of the endpoint ellipses. It is evident from this figure that for the position-grouped endpoints the ellipses are more or less circular, whereas for the vector-grouped endpoints the ellipses are elongated in the movement direction. This pattern was also reported by Hudson and Landy (2012) and corresponds to averaging ellipses oriented along the direction of movement.

**Fig. 5:**
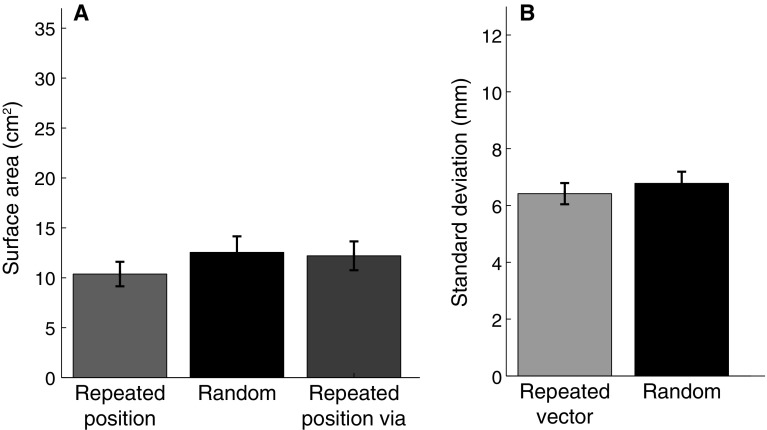
Results of the main experiment, averaged across the 12 participants. *Error bars* represent the SEM between participants. **a** Surface area of the 95% confidence ellipses of movement endpoints for position-grouped data. The surface area for the repeated vector block is 11.9 cm^2^ (not shown in the figure). **b** Standard deviation in the movement endpoints orthogonal to the movement direction for vector-grouped data

To test whether participants benefited from repeating a position, we compared the precision (averaged across endpoints) between the repeated position block and the random block (Fig. 5a). We did not find the predicted consistent difference between the surface area of the 95% confidence ellipses for the repeated position block and the random block (*t*_11_ = 1.20, *p* = 0.23). The small tendency (17%) in the predicted direction might be related to making back-and-forth movements, because the difference between the repeated position via block and the random block was even smaller (3%) and more clearly not significant (*t*_11_ = 0.21, *p* = 0.83).

To test whether participants benefited from repeating a vector, we compared the standard deviations in the direction orthogonal to the movement direction in the repeated vector block and the random block (Fig. 5b). Although there was a slight tendency in the predicted direction (5%), this tendency was far from significant (*t*_11_ = 0.97,* p* = 0.35).

## Control experiment

In our main experiment, we found no systematic differences between the endpoint errors when repeating a position, repeating a vector or not repeating either of them. There was a tendency for the variability to be smaller in the blocks with repetitions, but the differences were small and not significant. In a previous study, Hudson and Landy (2012) found that participants clearly benefited from repeating a position or a vector. In their study, participants moved in a different region than where the targets and the feedback were presented, so differences in variability could arise from adjustments to the visuo-motor mapping, rather than from adjustments to the movements themselves.

**Fig. 6:**
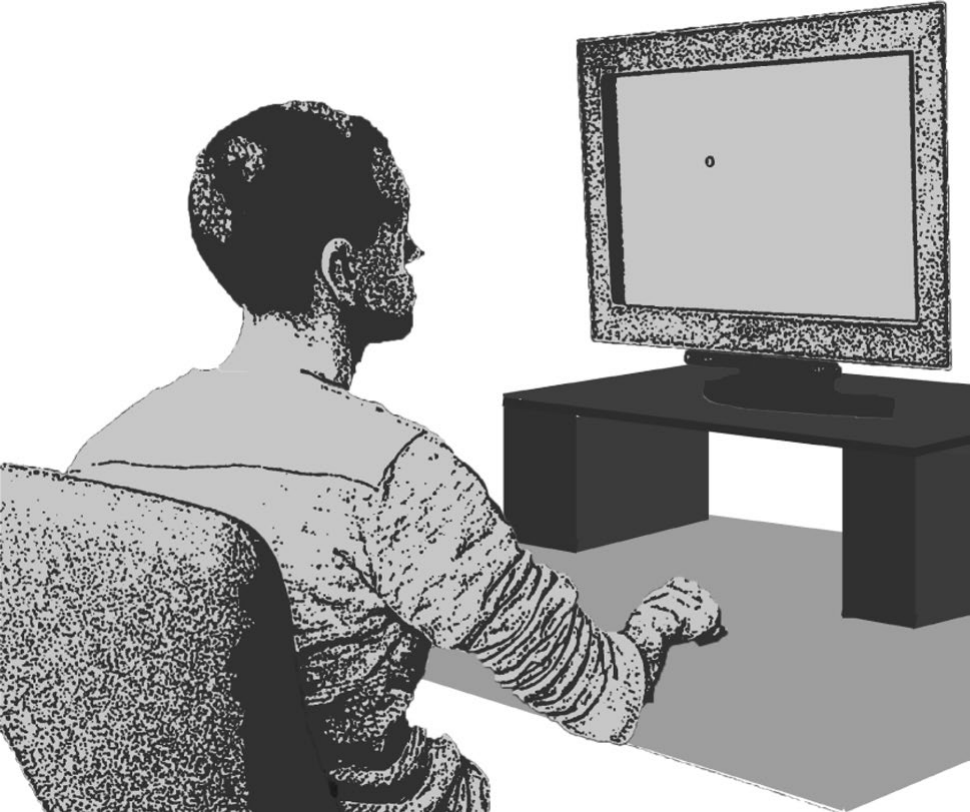
Experimental setup of the control experiment. Movements were recorded with an Optotrak system, which was placed approximately 2 m behind the computer screen

To check whether we can attribute all the differences between our results and those of Hudson and Landy to the transformation between the table and the screen, we repeated our experiment with 12 new participants (but skipping the repeated position via block). Instead of using the mirror setup, the participants were now seated in front of a table with a monitor in front of them (Fig. 6). The participants saw the targets on the computer screen and performed the movements on the table. The dimensions of the image on the computer screen were exactly the same as those on the horizontal screen in the main experiment. In all other respects, the experiment was also identical to the main experiment.

## Results

**Fig. 7:**
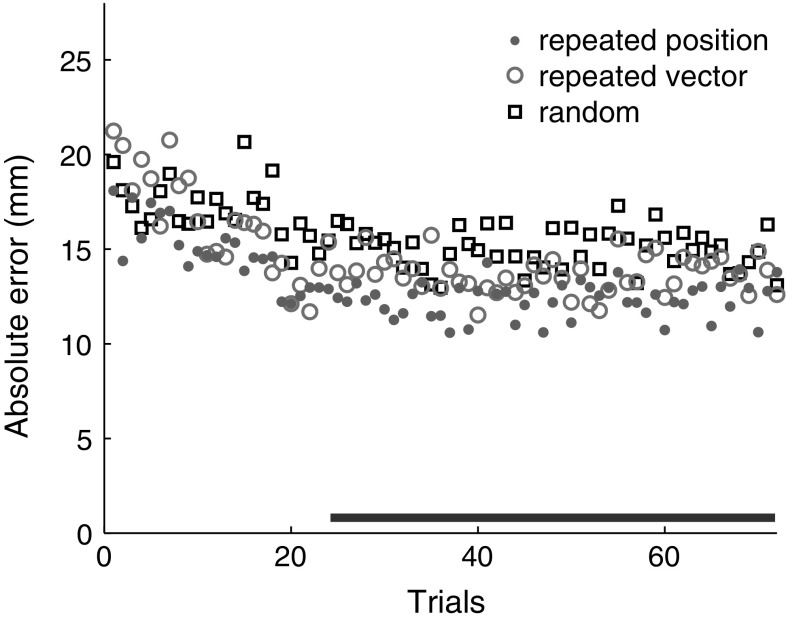
Absolute errors in the control experiment, averaged as in Fig. 3

**Fig. 8:**
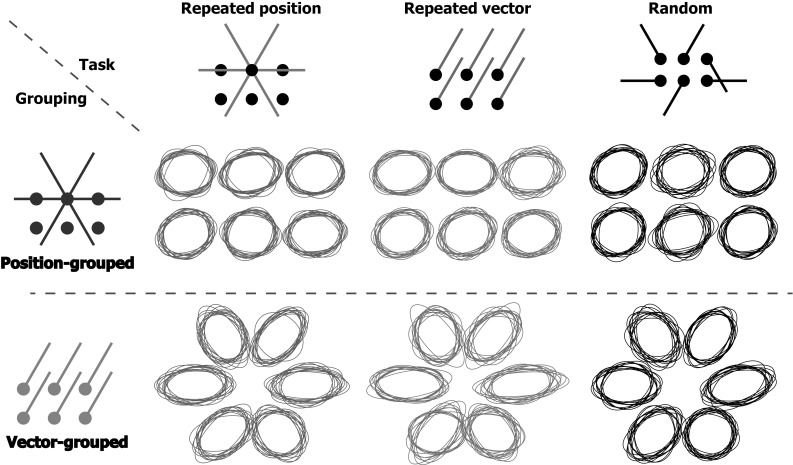
95% confidence ellipses for the different blocks (*columns*) and types of grouping (*rows*) for the control experiment. For further details, see Fig. 4

Average movement times were 266 ms, irrespective of the block that was performed. Figure 7 shows that the absolute errors were larger than in the main experiment, but again any refinements to the movements occurred within the first 24 trials. The errors were smaller in the repeated position block than in the repeated vector block, and smaller in both these blocks than in the random block. As in the main experiment, the behaviour was consistent across participants (Fig. 8).

**Fig. 9:**
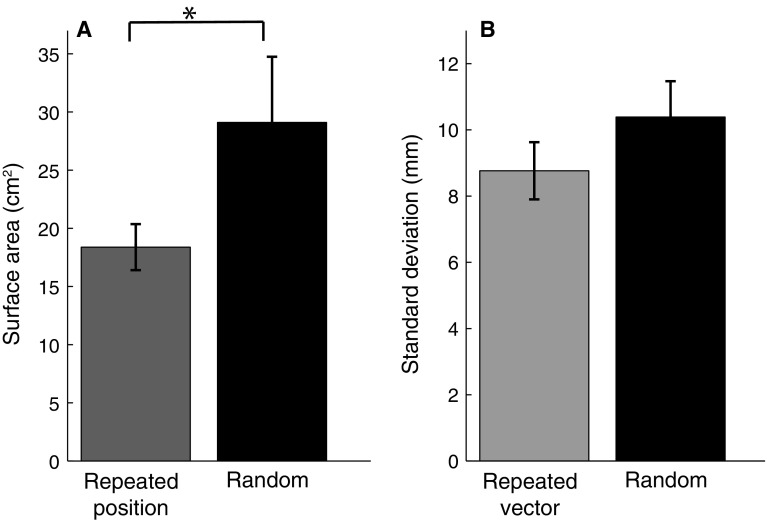
Results of the control experiment, averaged across the 12 participants. *Error bars* represent the SEM between participants. **a** Surface area of the 95% confidence ellipses of position-grouped data. Surface area for the repeated vector block is 23.1 cm^2^ (not shown in the figure) **b** Standard deviation in the direction orthogonal to the movement direction for vector-grouped data

To test whether participants benefit from repeating a position in this experiment, we compared the precision (averaged across all endpoints) in the repeated position block with that in the random block (Fig. 9a). In line with the prediction, the surface area of the repeated position block was significantly smaller (37%) than the surface area of the random block (*t*_11_ = 2, 42, *p* < 0.05).

To test whether participants benefit from repeating a vector, we determined the standard deviations in the direction orthogonal to the movement direction for movements in the same direction. We compared the individual averages of these values for the repeated vector block with those in the random block (Fig. 9b). The difference was in the predicted direction, but was small (15%) and not significant (*t*_11_ = 1, 56, *p* = 0.15).

## Discussion

In this study, we compared identical movements that had a different history. We compared blocks in which the same position was repeated and blocks in which the same vector was repeated. These two blocks were compared with a block in which neither a position nor a vector was ever repeated. We found that when participants had to move in a different region than where the target and feedback were provided (as in using a computer mouse; control experiment), they consistently benefited from repeating a position, in line with the results of Hudson and Landy (2012). This makes sense, because proprioceptive memory of the position can help localize it on the next trial. When participants moved their hand to the visible target position itself (main experiment), the benefit of repeating the same position was negligible. In neither case did participants’ precision in the direction orthogonal to the movement direction (i.e. in the direction of motion) increase consistently when a vector was repeated.

The shapes of the confidence ellipses might tell us something about how participants code their movements. Hudson and Landy (2012) based their conclusion that position is coded in repeated position blocks on the fact that participants’ movement endpoints had round confidence ellipses in such block, even if the movements were vector-grouped. We found that the vector-grouped confidence ellipses were elongated in the direction of movement, both for the main experiment and for the control experiment that was a replication of their study. Other studies also find movement endpoint distributions that are elongated in the movement direction when repeating movements from the same starting point to the same target with target and hand co-located as in our main experiment (van Beers et al. 2004) and when moving from a single starting point to various targets presented in random order with a vertical computer screen as in our control experiment (Gordon et al. 1994).

**Fig. 10:**
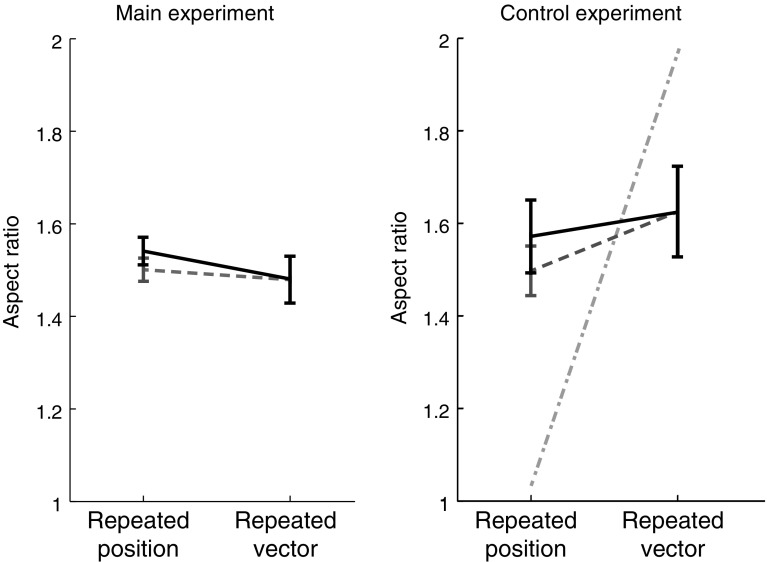
Average aspect ratio of the 95% confidence ellipses (long divided by short axis length) for the vector-grouped data of both experiments, with our way of averaging (*dark solid line*) and Hudson and Landy’s way of averaging (*light dotted line*). For comparison, we added the results of Hudson and Landy (2012) by a *green dash-dotted line*. *Error bars* represent the SEM across participants (color figure online)

What might be the reason that the shapes of the confidence ellipses in our control experiment, which was a close replication of Hudson and Landy’s (2012) study, differ from those of that study? One difference between the two studies is that we averaged our data in a different way than they did. As we were only interested in the variability, we removed systematic errors separately for each start–target combination. This correction was the same for position grouping as for vector grouping. Hudson and Landy did not remove systematic errors separately for each movement direction when analysing the repeated position block. If we perform the analysis on our data in their manner, we find that the results for the repeated position block (this aspect of the analyses only differed for that block) are slightly more similar to the results of Hudson and Landy (they found an aspect ratio of 1.03 for the repeated position block, see green line in Fig. 10). We see that in both experiments the aspect ratio is closer to one when conducting the analysis in the same way as Hudson and Landy did so, than it is for our analysis (Fig. 10). However, the aspect ratios remain quite similar for the two blocks and are clearly larger than one for the repeated position block (meaning that the ellipses are clearly elongated). Thus, although a small part of the difference between our and their results can be attributed to the different way of analysing the data, this cannot be the whole explanation.

Another experimental difference is that Hudson and Landy’s (2012) rewarded the subjects more explicitly, by showing exploding targets. In our experiment, the reward was only implicit: subjects could see the size of their error. As what is learned can differ between reward-based learning and error-based learning (Huang et al. 2011; Galea et al. 2015; Dayan et al. 2014), such a minor experimental detail might give rise to quite different results.

Vector and position coding might not be the only ways of planning. It has also been suggested that a whole trajectory is planned (Scott and Kalaska 1995). In the repeated position block of this study, not only the position is repeated, but also the trajectory is the reverse of the one that brought the hand to the starting position. Therefore, the trajectory to the starting position could also have provided information that participants used to make more accurate movements. In our main experiment, participants tended to be slightly more precise in the repeated position block than in the repeated vector or in the random block (although the effect is not statistically significant). This tendency was gone when participants were guided to the start position along a curved trajectory (Fig. 5a). There is therefore some (non-significant) indication that participants might use trajectory information to improve their precision in the repeated position block, but note that neither this nor the effects of repeating a position or movement direction were significant when moving to visually perceived positions without any additional transformation in the main experiment.

## Conclusion

When moving in a different region than where the targets are seen and feedback is provided, participants benefit from repeating a position, but not from repeating a vector. When moving to the actual visible targets with feedback provided at the position of the hand, there was no consistent benefit of repeating a position or a vector. This could mean that fine-tuning the endpoint is particularly useful when dealing with unusual visuo-motor mappings, for instance by remembering the felt position of the hand at the endpoint to help bring the virtual position of the hand (cursor) to the target on the screen when the same endpoint is repeated across trials.
